# Value of proteinuria in evaluating the severity of HELLP and its maternal and neonatal outcomes

**DOI:** 10.1186/s12884-023-05862-5

**Published:** 2023-08-18

**Authors:** Yan Jiao, Yan Liu, Hongyuan Li, Zimeng Song, Shiliang Wang, Jiao Zhang, Jian Li, Jia Liu, Peng Wang, Yanhong Chen

**Affiliations:** 1 Department of obstetrics and gynecology of Xuzhou Central Hospital, No.199, Jiefang South Road, Xuzhou, Jiangsu, 221009 People’s Republic of China; 2 Department of Clinical Laboratory, Xuzhou Central Hospital, No.199, Jiefang South Road, Xuzhou, Jiangsu, 221009 People’s Republic of China; 3 Department of neurology, Xuzhou Central Hospital, No.199, Jiefang South Road, Xuzhou, Jiangsu, 221009 People’s Republic of China; 4 School of medical technology, Xuzhou Medical University, Xuzhou No.209, Tong Shan Road, Xuzhou, 221000 Jiangsu People’s Republic of China

**Keywords:** Proteinuria, HELLP, Severity of illness index, Pregnancy complications, Pregnancy outcome

## Abstract

**Background:**

HELLP syndrome refers to a group of clinical syndromes characterized by hemolysis, elevated liver enzymes and low platelet, and the evidence on the association between proteinuria and the severity of HELLP and its maternal and neonatal outcomes is rare.

**Methods:**

106 pregnant women were assigned to the proteinuric group (24-hUPro ≥ 0.3 g, 79 cases) and the non-proteinuric group (24-hUPro < 0.3 g, 27 cases). The proteinuric group was further divided into three subgroups: mild group (24-hUPro:0.3-2.0 g, 33 cases), moderate group (24-hUPro:2.0-5.0 g, 21 cases) and severe group (24-hUPro: ≥5.0 g, 25 cases). The general clinical data, laboratory indexes, complications and pregnancy outcome and adverse neonatal outcomes of HELLP with or without proteinuric were analyzed.

**Results:**

Compared with proteinuric group, the non-albuminuric group or in the three proteinuric subgroups of HELLP pregnant women’s, increased proteinuria was associated with earlier onset gestations, higher incidence of abdominal pain, skin jaundice, headache, blurred vision (*p* < 0.05 respectively), and also the higher levels of ALT, AST, LDH, Fib, APTT, ATII, proportions of tubular urine and lower levels of ALB, PLT (*p* < 0.05 respectively). In the three subgroups of the proteinuric group, the ratio of fetal growth restriction, cesarean section and postpartum hemorrhage were compared, and the difference was statistically significant (*p* < 0.05 respectively). Compared with the proteinuric group, the non-proteinuric group had higher birth weight, birth length, and lower SGA, admission rate in NICU (*p* < 0.05 respectively). In the three subgroups of the proteinuric group, significant differences were identified in the adverse outcomes of newborns (*p* < 0.05 respectively), and the incidence of adverse outcomes in neonates tended to be higher. Significant differences were identified in birth weight, birth length, and lower SGA and NICU occupancy rate among the three subgroups (*p* < 0.05 respectively).

**Conclusions:**

HELLP syndrome is a severe complication of pregnancy, involving multiple systems of the whole body. It has posed a great challenge to obstetricians for its acute onset, dangerous condition, rapid progress, and great harm. Thus, insights into HELLP syndrome should be gained, and early diagnosis, early treatment and timely termination of pregnancy should be conducted to reduce the incidence of maternal and fetal adverse outcomes and improve maternal and fetal prognosis.

## Background

HELLP syndrome refers to a group of clinical syndromes characterized by hemolysis, elevated liver enzymes and low platelet. It is a serious multi-system disease during pregnancy. The incidence of HELLP syndrome in pregnancy is nearly 0.5%~0.9%, which can occur at any time from pregnancy (70%) to several days after delivery (30%). It is most common between 27 ~ 37 weeks of pregnancy, and a few cases (10%) occur before 27 weeks of pregnancy [1, 2]. The pathological mechanism of the HELLP is not yet clear, which may be correlated with autoimmunity [3], placental origin [4], the antiphospholipid antibody syndrome (APLS) [5], and so on. Studies show that sisters and children of women with HELLP syndrome are at increased risk [6, 7]. Women with a history of HELLP pregnancy have a high risk of HELLP and PE in subsequent pregnancies [8–10].

The classification of HELLP syndrome follows two main diagnostic definitions: Tennessee or Mississippi Triple Class System, and Tennessee has been extensively employed for diagnosis [11]. In the Tennessee classification, concentrations of lactate dehydrogenase (LDH) was adopted to evaluate hemolysis, aspartate- and alanine-aminotransferase (AST, ALT) to liver injury and platelet counts to low platelet counts, respectively. The Mississippi classification stresses the severity of HELLP based on the lowest platelet count [12]). The clinical manifestations of HELLP syndrome are diverse, develop rapidly, and significantly jeopardize the health of mothers and infants. Delayed treatment often endangers the lives of mothers and infants, and maternal and infant complications and mortality are high [13]. The maternal mortality rate is 3.4–24.2%, and the perinatal mortality rate is as high as 7.7–60.0% [14].

Although HELLP syndrome has been considered a serious form of preeclampsia, a disease with pregnancy induced hypertension and proteinuria, some research systems are counteracting this view since HELLP is only correlated with hypertension and/or albuminuria in 80% of cases and exhibits different cytokine activation and aggravating placental angiopathy [15–18]. Moreover, the pathogenesis of the syndrome is complex and remains unclear, such that more specific/sensitive laboratory standards should be developed for the latest HELLP screening, diagnosis, and treatment. On that basis, early detection and timely and effective intervention take on a critical significance. In this study, the clinical characteristics, pregnancy outcomes and risk factors of HELLP syndrome in pregnant women with different 24hUPro levels were analyzed retrospectively to provide reference data for preventing and reducing of HELLP syndrome.

## Materials and methods

### Study subjects

A total of 127 pregnant women with HELLP syndrome were selected from Xuzhou Central Hospital between January 2012 and December 2021 as the study subjects, of which 21 were excluded due to lack of the quantitative results of 24-hUPro. The remaining 106 were included in this study. 106 pregnant women were assigned to the proteinuric group (24-hUPro ≥ 0.3 g) and the non-proteinuric group (24-hUPro < 0.3 g). The proteinuric group was further divided into three subgroups: mild group (24-hUPro:0.3-2.0 g), moderate group (24-hUPro:2.0-5.0 g) and severe group (24-hUPro: ≥5.0 g). Patients with HELLP syndrome should conform to the diagnostic criteria of Tennessee [19]. Patients suffering from hepatitis, any comorbidities such as lupus or other immune disorders, tumors, intrahepatic cholestasis of pregnancy, biliary tract disease, ischemic hepatitis due to postpartum hemorrhage, and gestational diabetes, patients with chronic hypertension or type 1 or 2 diabetes, patients with incomplete clinical data were excluded. This study gained approval from the Hospital Ethics Committee of Xuzhou Central Hospital [XZXY-LJ-20110110-079], and all methods were performed in accordance with the relevant guidelines and regulations. Written informed consent was gained from all patients.

### Data collection

Data Collection: using case report form to collect data as follows: (1) general information: age, body mass index (BMI), gestational age, number of pregnancies, parity, number of pregnancies, blood pressure at admission and diagnosis at admission; (2) clinical symptoms; (3) laboratory indexes within 24 h after admission: alanine aminotransferase (ALT), aspartate aminotransferase (AST), albumin (ALB), total bilirubin (TBil), direct bilirubin (DBil), alkaline phosphatase (ALP), γ-glutamyl transpeptidase (γ-GGT), total bile acid (TBA), lactate dehydrogenase (LDH), white blood cell count (WBC), hemoglobin (Hb), platelet count (PLT), prothrombin time (PT), international normalized ratio (INR), fibrinogen (FIB), activated partial thromboplastin time (APTT), antithrombin III (ATIII), serum creatinine (Scr), uric acid (UA), blood urine nitrogen (BUN), tubular urine; (4) complications; (5) maternal and infant outcomes.

### Statistical analysis

Variables with normal distribution are expressed as mean ± standard deviation, the differences between two groups were examined through an independent samples t-test, over two groups were compared through one-way ANOVA. Moreover, variables with skewed distribution are expressed as median with inter-quartile range (IQR), comparisons between groups were analyzed through Mann-Whitney U test and Kruskal-Wallis H test. Categorical variables are presented as frequencies with percentages and compared via the Pearson chi-square test. Additionally, the receiver operating characteristic curve (ROC) and area under curve (AUC) were used as the assessment indicators of sensitivity and specificity of HELLP biomarkers for proteinuric and other clinical laboratory indicators analysis. A two-sided p-value of less than 0.05 meant a statistically significant difference. All statistical analysis was conducted using the SPSS 25.0 software (IBM, USA).

## Results

### Comparison of general clinical data of pregnant women with HELLP in the respective group

A total of 106 pregnant women with HELLP were recruited in this study. The 24-hUPro levels ranged from 0.1 to 21.3 g, with 27 cases (25.47%, 27/106) in the non-proteinuric group and 79 cases (74.53% ,79/106) in the proteinuric group. Moreover, in the proteinuric group, there were 33 cases in the mild group, 21 cases in moderate group and 25 cases in the severe group (Table 1).

**Table 1:** Comparison of general clinical data of HELLP pregnant women in the respective group

Factors	nonproteinuric group (n = 27)	proteinuric group (n = 79)	*P*	proteinuric group			
				mild group (n = 33)	moderate group (n = 21)	severe group (n = 25)	*P*
Age (years old)	31.0 (28.0–33.0)	31.0 (29.0–34.0)	0.221	32.0 (29.0–35.0)	31.0 (27.5–34.5)	31.0 (29.0-33.5)	0.659
Gestational age at onset (week)	38.0 (37.0–38.0)	34.0 (32.0–36.0)	0.000	36.0 (35.0-36.5)	34.0 (33.0–36.0)	30.0 (28.0-31.5)	0.000
Gestational age at termination of pregnancy (week)	39.0 (38.0–39.0)	36.0 (34.0–37.0)	0.000	37.0 (36.5–38.0)	35.0 (34.0–36.0)	33.0 (32.0–35.0)	0.000
BMI	26.4 (24.3–27.5)	26.1 (23.2–27.6)	0.227	26.1 (22.6–28.6)	25.7 (23.3–27.5)	26.1 (23.1–27.3)	0.978
Pregnant Times (n)	2 (1–3)	2 (1–3)	0.979	2 (1–3)	2 (1–3)	2 (1–3)	0.876
Parity	1 (1–2)	1 (1–2)	0.927	1 (1–1)	1 (1–2)	1 (1–2)	0.231
Twins (n %)	2 (7.41)	4 (5.06)	0.669	1 (3.03)	2 (9.52)	1 (4.00)	0.546
Preeclampsia /Eclampsia	27 (100)	78 (98.73)	0.557	32 (96.97)	21 (100)	25 (100)	0.494
History of hypertension (n %)	0 (0)	1 (1.27)	0.557	0 (0)	0 (0)	1 (4.00)	0.000
SBP (mmHg)	154 (148–159)	156 (149–162)	0.238	150 (147–157)	157 (154–160)	162 (155–165)	0.000
DBP (mmHg)	102 (98–108)	102 (95–107)	0.410	98 (90–102)	102 (96–107)	108 (102–112)	0.000
Clinical symptoms (n %)							
Nausea, vomiting	4 (14.81)	13 (16.46)	0.841	3 (9.09)	3(14.29)	7 (28.00)	0.150
Abdominal pain	0 (0)	5 (6.33)	0.181	0 (0)	2 (9.5)	3 (12.00)	0.000
Skin jaundice	0 (0)	1 (1.27)	0.557	0 (0)	0 (0)	1 (4.00)	0.000
Headache	2 (7.41)	13 (16.46)	0.244	2 (6.06)	3 (14.29)	8 (32.00)	0.029
Blurred vision	1 (3.70)	15 (18.99)	0.055	2 (6.06)	4 (19.05)	9 (36.00)	0.016
Edema	8 (29.63)	38 (48.10)	0.095	11 (33.3)	10 (47.62)	17 (68.00)	0.000

Compared with proteinuric group, the non-albuminuric group HELLP pregnant women’s diagnosis and delivery gestational week is later (*p* < 0.05 respectively). No significant differences were identified in age, BMI before pregnancy, the proportion of twins, preeclampsia/eclampsia, blood pressure, headache and nausea, vomiting, abdominal pain, skin jaundice, blurred vision, edema between the two groups (*p* > 0.05 respectively).

Significant differences were identified in gestational age at diagnosis, gestational age at termination of pregnancy, history of hypertension, SBP, DBP (*p* < 0.05). The incidences of abdominal pain, skin jaundice, headache, blurred vision, edema in severe HELLP group were significantly higher than those in mild and moderate HELLP groups (*p* < 0.05), no significant difference was identified in other symptoms between the subgroups (*p* > 0.05) (Table 1).

### Comparison of the results of HELLP pregnant women’s laboratory indexes

Compared with the non-albuminuric group, the HELLP pregnant women of the proteinuric group has the higher levels of ALT, AST, ALB, LDH, PLT, FIB, APTT, and proportions of tubular urine (*p* < 0.05 respectively); Of note, the differences in the above indicators were also statistically significant in the three subgroups of the proteinuric group (*p* < 0.05 respectively), that is, with increasing urinary protein levels of HELLP pregnant women, ALT, AST, LDH, PLT, Fib, APTT, proportions of tubular urine tend to increase, while ALB tends to do the opposite. At the same time, other laboratory indexes such as TBil, DBil, ALP, γ-GGT, TBA, WBC, Hb, INR, BUN, SCr, UA were not significantly different between the non-albuminuric group and the albuminuric group, and the three subgroups of the albuminuric group (*p* > 0.05 respectively). As indicated by the above results, hemolysis, liver enzymes and thrombocytopenia occurred in HELLP patients with the increase of proteinuric, consistent with the typical symptoms of HELLP patients. There was a significant difference in PT level among the three subgroups (*p* = 0.015), but no significant difference was found in the comparison between the albuminuric group and the non-albuminuric group (*p* = 0.363) (Table 2).

**Table 2:** Comparison of laboratory test results of HELLP in pregnant women in the respective group

Factors	nonproteinuric group (n = 27)	proteinuric group (n = 79)	*P*	proteinuric group			
				mild group (n = 33)	moderate group (n = 21)	severe group (n = 25)	*P*
ALT (U/L)	122.0 (104.0-135.0)	139.0 (122.0-153.0)	0.003	132.0 (117.5-139.5)	141.0 (113.5–147.0)	162.0 (137.5–184.0)	0.001
AST (U/L)	152.0 (140.0-169.0)	168.0 (149.0-188.0)	0.017	153.0 (140.5–168.0)	173.0 (150.5–186.0)	187.0 (173.0-204.0)	0.000
ALB (g/L)	32.1 (28.6–34.4)	29.1 (26.6–31.7)	0.003	31.4 (28.8–33.1)	29.7 (27.5–31.7)	26.5 (24.6–27.6)	0.000
TBil (µmo/L)	15.4 (15.4–16.6)	15.6 (13.2–17.2)	0.526	15.6 (13.2–17.3)	15.1 (13.2–17.3)	15.7 (13.5–16.9)	0.967
DBil (µmo/L)	6.9 (5.3–7.4)	6.4 (5.4–7.4)	0.406	6.4 (5.4–7.9)	6.3 (5.4–7.2)	6.3 (4.9–7.5)	0.846
ALP (U/L)	122.2 (97.5-162.8)	124.3 (97.3-152.1)	0.698	110.4 (89.4-164.8)	138.3 (102.8-163.7)	124.3 (96.6-141.1)	0.514
γ-GGT (U/L)	44.0 (30.0–48.0)	39.0(27.0–51.0)	0.572	35.0 (25.5–45.5)	39.0(29.5–50.0)	43 (28.5–53.0)	0.473
TBA (µmo/L)	6.8 (4.7–9.2)	6.1 (4.4-8.0)	0.685	5.2 (3.8-8.0)	6.8 (4.6-8.0)	6.6 (5.1–8.1)	0.285
LDH (U/L)	347.0 (292.0-392.0)	432.0 (339.0-534.0)	0.000	339.0 (292.0-399.0)	453.0 (382.5-567.5)	552.0 (468.5-599.5)	0.000
WBC (×10^9^/L)	11.4 (9.1–13.5)	11.3 (8.9–12.8)	0.934	12.1 (9.7–13.5)	10.4 (8.1–12.6)	11.3 (8.9–13.1)	0.249
Hb (g/L)	126.0 (117.0-136.0)	127.0 (119.0-134.0)	0.919	127.0 (119.0-134.0)	127.0 (117.0-138.0)	127.0 (120.0-135.0)	0.930
PLT (×10^9^/L)	104.0 (98.0-112.0)	92.0 (85.0–97.0)	0.000	96.0 (92.5–105.0)	89.0 (84.0–96.0)	85.0 (78.0-89.5)	0.000
PT (s)	12.1 (11.1–12.9)	12.4 (11.4–13.2)	0.363	11.8 (11.1–12.5)	12.3 (11.9–13.2)	12.9 (12.1–13.8)	0.015
INR	1.3(1.1–1.5)	1.2 (0.9–1.4)	0.308	1.1 (0.9–1.3)	1.3 (1.0-1.5)	1.2 (1.0-1.5)	0.242
Fib (g/L)	2.7 (3.3–4.3)	4.2 (3.6–4.8)	0.014	3.8 (3.5–4.5)	4.3 (3.8–4.9)	4.5 (3.8–5.1)	0.031
APTT (s)	36.4 (33.5–40.3)	39.8 (35.7–45.3)	0.022	38.3 (32.7–43.3)	40.6 (37.4–44.6)	43.8 (38.0-49.7)	0.018
ATIII (%)	90.2 (84.2–93.6)	81.7 (75.3–87.9)	0.001	88.1 (83.2–94.0)	81.6 (77.3–87.0)	74.2 (70.6–79.2)	0.000
BUN (mmol/L)	6.3 (5.2–6.6)	6.4 (4.7–7.2)	0.514	6.4 (4.9–7.2)	6.4 (4.6–7.1)	6.3 (5.0-7.8)	0.617
SCr (µmo/L)	68.0 (55.0–78.0)	66.0 (55.0–84.0)	0.717	64.0 (52.5–79.0)	64.0 (57.5–91.5)	71.0(54.5–96.5)	0.299
UA (µmo/L)	250.0 (187.0-292.0)	268 (1780.-329.0)	0.135	257 (187.0-310.0)	285.0(212.0-339.5)	262.0 (184.5–340.0)	0.700
tubular urine (n %)	0 (0)	34 (43.04)	0.000	4 (12.12)	12 (57.14)	18 (72.00)	0.000

### Comparison of the complications and pregnancy outcome of HELLP pregnant women in the different groups

In the non-proteinuria group, 2 case (7.41%) had placental abruption, 5 cases (18.52%) had fetal growth restriction. There was no significant difference in total length of stay, proportion of ICU stay, length of ICU stay, the incidence of severe complications during pregnancy and delivery, cesarean section, abnormal blood pressure and maternal mortality between the non-proteinuric group and the proteinuric group (*p* > 0.05 respectively) (Table 3).

**Table 3:** Comparison of complications and pregnancy outcomes of HELLP pregnant women in the respective group

Factors	nonproteinuric group (n = 27)	proteinuric group (n = 79)	*P*	proteinuric group			
				mild group (n = 33)	moderate group (n = 21)	severe group (n = 25)	*P*
total length of stay (d)	9.48 ± 2.33	9.20 ± 2.33	0.593	8.91 ± 2.35	9.24 ± 2.57	9.56 ± 2.14	0.579
ICU stay (n %)	5 (18.52)	16 (20.25)	0.845	5 (15.15)	5 (23.81)	6 (24.00)	0.633
length of ICU stay (d)	3.13 ± 0.96	2.60 ± 0.89	0.292	3.2 ± 0.84	3.40 ± 1.34	3.33 ± 1.03	0.957
DIC (n %)	0 (0)	1 (1.27)	0.557	0 (0)	0 (0)	1 (4.00)	0.335
coagulopathy (n %)	0 (0)	2(2.53)	0.404	0 (0)	1 (4.76)	1 (4.00)	0.473
acute liver failure (n %)	0 (0)	2 (2.53)	0.404	0 (0)	1 (4.76)	1 (4.00)	0473
acute renal failure (n %)	0 (0)	3 (3.80)	0.304	0 (0)	1 (4.76)	2 (8.00)	0.277
pulmonary edema (n %)	0 (0)	1 (1.27)	0.557	0 (0)	0 (0)	1 (4.00)	0.335
placental abruption (n %)	2 (7.41)	8 (10.13)	0.676	1 (3.03)	3(14.29)	4 (16.00)	0.205
fetal growth restriction (n %)	5 (18.52)	21 (26.58)	0.401	4 (12.12)	6 (23.81)	11 (44.00)	0.021
fetal death in utero (n %)	0 (0)	2 (2.53)	0.404	0 (0)	0 (0)	2 (8.00)	0.109
cesarean section (n %)	21 (77.78)	64 (81.01)	0.716	23 (69.70)	17 (80.95)	24(96.00)	0.041
postpartum hemorrhage (n %)	4 (14.81)	14 (17.72)	0.728	1 (3.03)	4 (19.05)	9 (36.00)	0.005
blood pressure at 42 days after delivery (mmHg)							
SBP	125 (121–129)	125 (121–131)	0.624	124 (120–129)	126 (124–131)	125 (121–135)	0.261
DBP	74 (70–80)	79 (72–82)	0.051	79 (72–83)	76 (69–81)	79 (75–83)	0.204
maternal mortality (n %)	0 (0)	1 (1.27)	0.557	0 (0)	0 (0)	1 (4.00)	0.335

In the three subgroups of the proteinuric group, the ratio of fetal growth restriction, cesarean section and postpartum hemorrhage were compared, and the difference was statistically significant (*p* < 0.05 respectively). The incidences of placental abruption, fetal death in utero and maternal mortality were increased with the increase of urinary protein level, but there was no significant difference because of the lower incidences (*p* > 0.05 respectively). No significant differences were identified in total length of stay, proportion of ICU stay, length of ICU stay, the incidence of severe complications during pregnancy and delivery such as DIC, coagulopathy, acute liver failure, acute renal failure, pulmonary edema and blood pressure at 42 days after delivery between the three subgroups (*p* > 0.05 respectively) (Table 3).

In this study, 1 case (0.94%, 1/106) of pregnant women with HELLP died. The pregnant women had no chronic disease history and did not have regular antenatal check-up during pregnancy. At the 38th week of pregnancy, the case had extremely elevated blood pressure (260/120 mmHg), chest tightness and wheezing, dizziness, headache, blindness, light perception only, fundus hemorrhage, no significant abnormalities in liver and kidney function and immune indexes. After admission, there was 1 case of live birth by emergency section through comprehensive evaluation and multi-disciplinary cooperation in diagnosis and treatment. Although the patient’s condition was significantly relieved, the fluctuation of blood pressure was (130–160) /(80–100) mmHg. The case had intermittent fever and unrelieved blindness, deep coma and no spontaneous breathing. The imaging results showed intracranial hemorrhage, which was broken into the hypothalamus. The family gave up further treatment.

### Comparison of adverse neonatal outcomes in pregnant women with HELLP

Compared with the proteinuric group, the non-proteinuric group had higher birth weight, birth length, and lower SGA, admission rate in NICU (*p* < 0.05). The incidences of other complications and adverse outcomes (e.g., neonatal infection, neonatal jaundice, and neonatal anemia) in the non-albuminuric group were lower than those in the albuminuric group, and the differences also achieved statistical significance (*p* < 0.05 respectively) (Table 4).

**Table 4:** Comparison of neonatal outcomes of HELLP pregnant women in the respective group

Factors	nonproteinuric group (n = 27)	proteinuric group (n = 79)	*P*	proteinuric group			
				mild group (n = 33)	moderate group (n = 21)	severe group (n = 25)	*P*
1 min Apgar score	10 (10 ~ 10)	10 (9 ~ 10)	0.319	10 (10 ~ 10)	10 (8 ~ 10)	10 (8 ~ 10)	0.001
Birth weight (g)	2720 (2600–3230)	2470 (2100–2960)	0.013	2750 (2500–3140)	2390 (2200–2635)	2040 (1750–2455)	0.000
Birth length (cm)	47 (45–49)	44 (41–48)	0.005	46 (43–49)	45 (42–48)	42 (39–43)	0.001
SGA (n %)	3 (10.34)	39 (49.37)	0.000	7 (21.21)	13 (61.90)	19 (76.00)	0.000
NICU admission (n %)	2 (6.90)	43 (54.43)	0.000	11 (33.33)	11 (52.38)	21 (84.00)	0.001
Sex of newborn (n %)							
Male	13 (64.29)	44 (59.04)	0.449	16 (19.28)	12 (14.46)	16 (19.28)	0.580
Female	16 (35.71)	39 (40.96)	0.448	13 (15.66)	12 (14.46)	14 (16.87)	0.891
Neonatal asphyxia (n %)	0 (0)	9 (11.39)	0.067	1 (3.03)	2 (9.52)	6 (24.00)	0.017
NRDS (n %)	0 (0)	13 (16.46)	0.024	2 (6.06)	3 (14.29)	8 (32.00)	0.029
NEC (n %)	0 (0)	7 (8.86)	0.109	0 (0)	3 (14.29)	4 (16.0)	0.062
Neonatal infection (n %)	0 (0)	20 (25.32)	0.004	3 (9.09)	6 (28.57)	11 (44.00)	0.009
Neonatal jaundice (n %)	0 (0)	16 (20.25)	0.011	4 (12.12)	6 (28.57)	6 (32.00)	0.291
Neonatal anemia (n %)	0 (0)	14 (17.72)	0.019	2 (6.06)	3 (14.29)	9 (36.00)	0.011
Perinatal mortality (n %)	0 (0)	1 (1.27)	1.000	0 (0)	0 (0)	1 (4.76)	0.335

In the three subgroups of the proteinuric group, significant differences were identified in the adverse outcomes of newborns (*p* < 0.05), and the incidence of adverse outcomes in neonates tended to be higher. Significant differences were identified in birth weight, birth length, and lower SGA and NICU occupancy rate among the three subgroups (*p* < 0.05) (Table 4).

### Predictive value of proteinuric

**Fig. 1:**
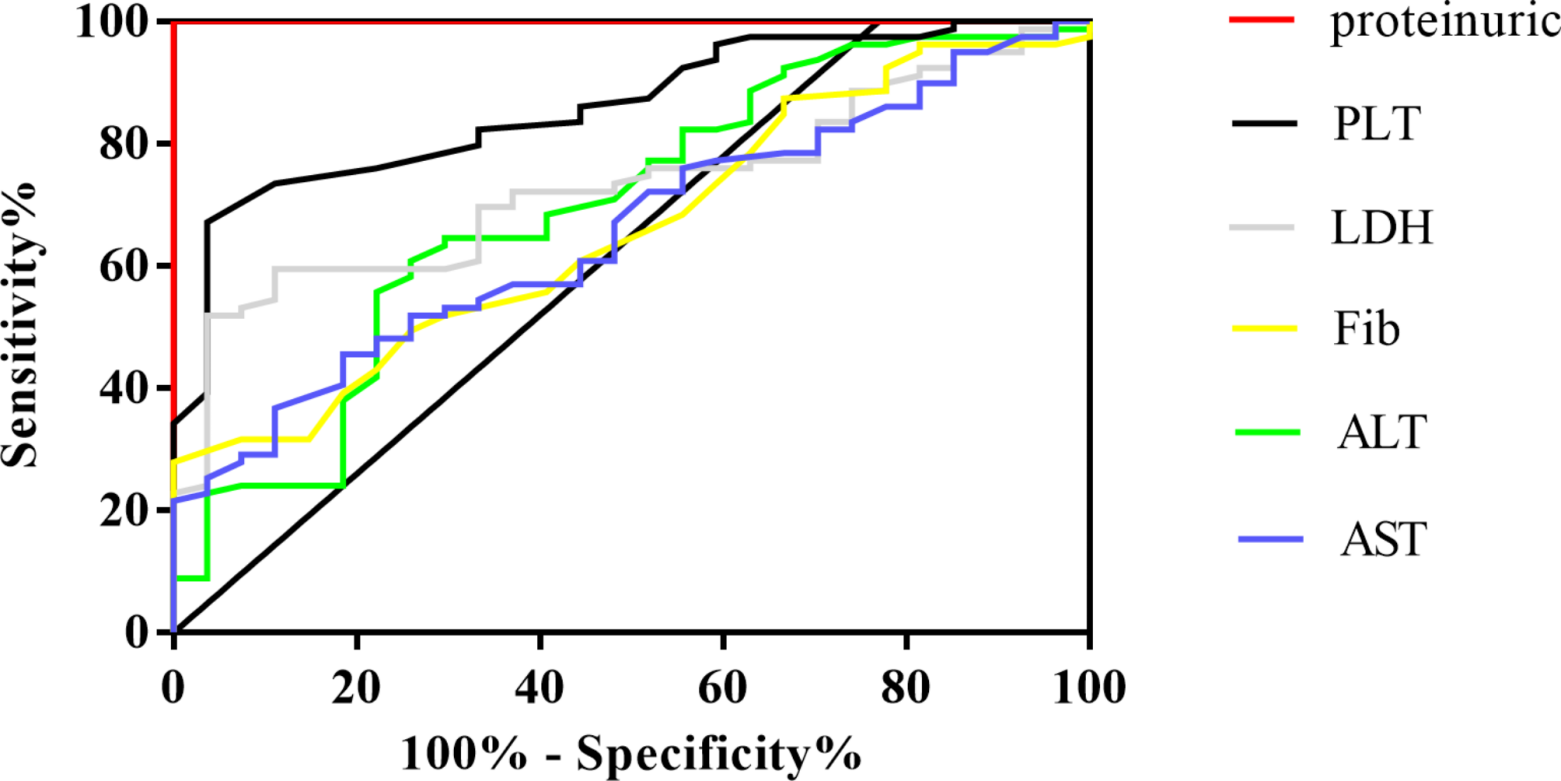
Receiver operating characteristic (ROC) analysis on the predictive capacity of the proteinuric (AUC = 1.00, 95% *CI*: 1.00–1.00, *P* < 0.001) and other clinical laboratory indicators for the occurrence of HELLP

The discriminative capability of proteinuric to determine HELLP was assessed using receiver-operator characteristic (ROC) curves. In this study, compared with non-proteinuric group, the the levels of proteinuric group of HELLP was showed significantly increased 0.17 (0.13–0.22) vs. 2.83(1.09–5.75) p < 0.001. In all subjects with HELLP, the area under the curve (AUC) for proteinuric was 1.00, 95% *CI*: 1.00–1.00, *P* < 0.001, the optimal cut-of value of proteinuric for HELLP detection in this study was > 0.38 g (sensitivity 98.73%, specificity 100.00%). And the AUC for PLT, ALT, LDH, Fib, AST was 0.86, 0.69, 0.73, 0.66, 0.65, 95% *CI*: 0.79–0.93, 0.57–0.81, 0.63–0.83, 0.55–0.77, 0.54–0.77, and *P* < 0.001, < 0.001, 0.002, 0.000, 0.014, 0.017 respectively (Fig. 1; Table 5).

**Table 5:** ROC analysis on the predictive capacity of proteinuric and other clinical laboratory indicators for the occurrence of HELLP.

variable	AUC	SE	*P*	95% *CI*	Sensitivity (%)	Specificity (%)
proteinuric	1.00	0.00	< 0.001	1.00–1.00	98.73	100.00
PLT	0.86	0.04	< 0.001	0.79–0.93	64.56	70.37
ALT	0.69	0.06	0.002	0.57–0.81	67.09	96.30
LDH	0.73	0.05	0.000	0.63–0.83	59.49	70.84
Fib	0.66	0.06	0.014	0.55–0.77	49.37	70.47
AST	0.65	0.06	0.017	0.54–0.77	45.57	81.48

## Discussion

Severe pre-eclampsia(PE) and HELLP syndrome put pregnant women at greater risk for life-threatining complications. The pathogenesis of PE is accompanied by glomerular lesions mediated by soluble vascular endothelial growth factor receptors, such as swelling of glomerular endothelial cells and relative occlusion of the capillary lumen, this change increases the tendency of renal microvascular thrombosis, resulting in cell damage, impaired glomerular filtration capacity, the emergence of proteinuria [20]. Some studies showed that the blood pressure, severe PE, headache, fetal growth restriction and abnormal umbilical blood flow increased with the increase of urinary protein level in all subgroups of proteinuria group. The indexes of hepatic and renal function and coagulation function also showed a tendency of deterioration with the increase of urinary protein, which indicated that the level of urinary protein reflected the severity of PE to some extent [21]. Therefore, the level of urinary protein reflects the characteristic renal damage during the development of PE, which is always one of the important indexes to evaluate the state of PE.

The main pathological changes of HELLP syndrome are similar to pre-eclampsia, it is characterized by systemic arteriosclerosis, endothelial cell injury leading to collagen exposure, coagulation activation, platelet activation and aggregation to form microvascular thrombosis, intravascular hemolysis, cholestasis, and then lead to liver, bile and other vascular system injury [22]. The pathogenesis of HELLP is accompanied by glomerular pathological changes mediated by soluble vascular endothelial growth factor receptors, such as swelling of glomerular endothelial cells and relative occlusion of capillary lumen, this change increases the tendency of renal microvascular thrombosis, resulting in cell damage, impaired glomerular filtration capacity, the emergence of proteinuria [23–25]. The main clinical manifestations of HELLP syndrome are right upper abdominal or upper abdominal pain, nausea, vomiting, general discomfort, and other non-specific symptoms, with hidden and atypical attacks. Nearly 15% of the cases have no hypertension or proteinuric [26]. In this study, the clinical data of HELLP pregnant women with different levels of 24-hUPro were retrospectively analyzed to explore the value of different levels of 24-hUPro in the evaluation of HELLP severity and maternal and fetal outcomes to facilitate better clinical monitoring and management of HELLP.

The pathogenesis of HELLP is accompanied by glomerulopathy mediated by soluble vascular endothelial growth factor receptor, exhibiting swelling of glomerular endothelial cells and relative occlusion of capillary lumen. This change increases the tendency of renal microvascular thrombosis, thus causing cell damage, impaired glomerular filtration capacity, and proteinuria [27–29]. Thus, the level of urinary protein reflects the characteristic renal damage during the development of HELLP, which is always one of the critical indexes to evaluate the state of HELLP.

The results indicated that in the respective subgroup of the albuminuric group, with the increase of urinary protein level, the blood pressure level of pregnant women and the incidence of severe symptoms such as abdominal pain, skin jaundice, headache, blurred vision, and edema increased; The indexes representing liver and kidney function and blood coagulation function also tended to be worsening with the increase of levels of 24-hUPro, thus suggesting that the 24-hUPro level reveals the severity of HELLP to a certain extent. An existing study has suggested that there was no difference in admission blood pressure, prenatal and postnatal peak blood pressure, and laboratory indicators among women with HELLP with different urinary protein levels [30]. The possible reason for this discrepancy is that the number of cases studied in this study was not sufficiently large, and the focus was different.

This study also found that HELLP patients with albuminuria also had significant differences in laboratory tests compared with those without albuminuria, such as ALT, AST and ALB which represent abnormal liver function, the difference of LDH, PLT, FIB, APTT and ATIII, which represent abnormal coagulation function, has clinical significance (*p* < 0.05 respectively). Of note, the differences in the above indicators were also statistically significant in the three subgroups of the proteinuric group (*p* < 0.05 respectively).

This study indicated that the incidence of fetal growth restriction, neonatal asphyxia, cesarean section, postpartum hemorrhage, and some other complications and adverse outcomes (e.g., neonatal infection, neonatal jaundice, and neonatal anemia) tended to increase with the increase of the urine protein level. Maternal death after delivery only occurred in the severe group, whereas the difference did not achieve statistical significance. The results of other studies over the past few years are basically consistent with this study [31]. Özkara [32] et al. analyzed the incidence of HELLP, placental abruption, eclampsia in pregnant women with different urinary protein levels, similar to the results of this study. Although the incidence of the above adverse outcomes in pregnant women with high urinary protein levels is higher than those with low urinary protein levels, the difference is not statistically significant. The results of a multi-center study of 2305 patients by Wu [33] revealed that no significant difference was identified in HELLP syndrome, heart failure, pulmonary edema, placental abruption, and cesarean section rate among PE women with different 24-hUPro levels. The reason for the above result may be correlated with the low number of pregnant women with the above serious adverse outcomes. In addition, the recognition, monitoring and treatment of HELLP by medical workers are very effective and timely, the risk of serious adverse outcomes with severe HELP is significantly avoided.

It is noteworthy that there was one maternal death case in this study. The clinical warning of this case has two points as follows. First, standardized perinatal management can effectively ensure pregnant women’s health, and regular antenatal check-up helps to identify the disease at the early onset stage and treat it as soon as possible for avoiding the disease continued to progress to a critical state and the loss of valuable time for treatment. Second, the critical HELLP pregnant women at the postpartum stage should continue to maintain attention, continuous evaluation and identification of the disease, always alert to postpartum eclampsia, hypertension emergencies, cardiovascular and cerebrovascular accidents, and other complications.

Although the correlation between urinary protein levels and adverse outcomes in pregnant women is still controversial, most studies have shown that urinary protein level is clearly correlated with adverse outcomes of newborns. Lei et al. [34] showed that low birth weight, preterm birth rate, and stillbirth rate were positively correlated with 24-hUPro levels. Tanacan et al. [35] have suggested that small for gestational age infants, neonatal asphyxia, neonatal respiratory distress syndrome (NRDS), intraventricular hemorrhage, and neonatal necrotizing enterocolitis (NEC) were more common in the large proteinuria group. The results also indicated that the higher the level of urinary protein, the higher the incidence of adverse neonatal outcomes will be. The above results suggest that the neonatal outcome of HELLP pregnant women may be correlated with the level of urinary protein. However, most adverse neonatal outcomes, such as birth weight, length, NICU admission, asphyxia, NRDS, NEC, etc., are correlated with the small gestational age of delivery. In clinical practice, some pregnant women will terminate their pregnancy because of the simple increase of severe urinary protein level. Accordingly, the adverse neonatal outcome is attributed to the considerable amount of proteinuria in preterm delivery, thus requiring follow-up prospective research for further discussion.

The researchers have classified the cases of preeclampsia with hemolysis, or elevated liver enzymes, or thrombocytopenia as atypical HELLP syndrome, thus suggesting that advanced age, multiple births, hepatitis B virus infection and obesity are potential risk factors for atypical HELLP syndrome [36]. HELLP refers to a disease involving multiple organ systems of the whole body. Besides the liver and kidney, which are the most frequently involved organs, cardiovascular, nervous, blood and other multiple systems will exhibit relevant pathophysiological changes [37]. In the atypical preeclampsia (e.g., pregnancy-induced hypertension with microangiopathy/hemolysis, proteinuria with microangiopathy/hemolysis, preeclampsia or preeclampsia or HELLP syndrome 48 h after delivery, preeclampsia or preeclampsia or Hellp syndrome before 20 weeks’ gestation), 96% of the patients were initially referred for vasospasm symptoms (e.g., headache, visual impairment, hearing impairment, and gastrointestinal symptoms). Besides, nearly one-third of the cases had fetal growth restriction, the decreased levels of placental growth factor (PLGF) were correlated with early onset preeclampsia and adverse pregnancy outcomes. As a relatively special HELLP, the diagnosis of protein-free HELLP is primarily dependent on the damage of other target organs, which is often accompanied by the abnormal performance of vital organs. The deterioration potential of its adverse outcome cannot be ignored due to the low level of urine protein. To achieve the objective of clinical postpartum management, aiming to improve the symptoms of puerperant women, HELLP syndrome will improve significantly in 48 h after delivery, such that the patient’s vital signs and various laboratory indicators should be closely monitored. Patients with postpartum rebound or aggravation of their condition and with postpartum HELLP syndrome should be given symptomatic treatment actively (e.g., aggressive blood pressure control, magnesium sulfate prevention of eclampsia, fluid and electrolyte management, glucocorticoid treatment, blood transfusion and even plasma exchange, and prevention of deep vein thrombosis embolism).

## Conclusion

In brief, HELLP syndrome is a severe complication of pregnancy, involving multiple systems of the whole body. It has posed a great challenge to obstetricians for its acute onset, dangerous condition, rapid progress, and great harm. Thus, insights into HELLP syndrome should be gained, and early diagnosis, early treatment and timely termination of pregnancy should be conducted to reduce the incidence of maternal and fetal adverse outcomes and improve maternal and fetal prognosis.

## Abbreviations

HELLP: hemolysis, elevated liver enzymes and low platelet
BMI: Body mass index
SBP: systolic blood pressure
DBP: diastolic blood pressure
ALT: alanine aminotransferase
AST: aspartate aminotransferase
ALB: albumin
TBil: total bilirubin
DBil: direct bilirubin
ALP: alkaline phosphatase
γ-GGT: γ-glutamyl transpeptidase
TBA: total bile acid
LDH: lactate dehydrogenase
WBC: white blood cell count
Hb: hemoglobin
PLT: platelet count
PT: prothrombin time
INR: international normalized ratio
FIB: fibrinogen
APTT: activated partial thromboplastin time
ATIII: antithrombin III
Scr: serum creatinine
UA: uric acid
BUN: blood urine nitrogen
DIC: disseminated intravascular coagulation
NICU: Neonatal intensive care unit
NRDS: Neonatal respiratory distress syndrome
NEC: Necrotizing enterocolitis
